# Global view of a drug-sensitivity gene network

**DOI:** 10.18632/oncotarget.23229

**Published:** 2017-12-14

**Authors:** Haixiu Yang, Yunpeng Zhang, Jiasheng Wang, Tan Wu, Siyao Liu, Yanjun Xu, Desi Shang

**Affiliations:** ^1^ College of Bioinformatics Science and Technology, Harbin Medical University, Harbin 150081, China

**Keywords:** drug, sensitivity gene, network, enrichment analysis, network analysis

## Abstract

An important challenge in drug development is to gain insight into the mechanism of drug sensitivity. Looking for insights into the global relationships between drugs and their sensitivity genes would be expected to reveal mechanism of drug sensitivity. Here we constructed a drug-sensitivity gene network (DSGN) based on the relationships between drugs and their sensitivity genes, using drug screened genomic data from the NCI-60 cell line panel, including 181 drugs and 1057 sensitivity genes, and 1646 associations between them. Through network analysis, we found that two drugs that share the same sensitivity genes tend to share the same Anatomical Therapeutic Chemical classification and side effects. We then found that the sensitivity genes of same drugs tend to cluster together in the human interactome and participate in the same biological function modules (pathways). Finally, we noticed that the sensitivity genes and target genes of the same drug have a significant dense distance in the human interactome network and they were functionally related. For example, target genes such as epidermal growth factor receptor gene can activate downstream sensitivity genes of the same drug in the PI3K/Akt pathway. Thus, the DSGN would provide great insights into the mechanism of drug sensitivity.

## INTRODUCTION

There is compelling evidence that predictions of anticancer drug response can be refined by identifying and applying molecular biomarkers [1, 2]. For example, the use of drugs to target the protein product of the *BCR–ABL* translocation in chronic myeloid leukemia, or the *BRAF* gene in malignant melanoma, helped transform the treatment of these diseases and substantially improve survival rates [3, 4]. In recent years, enormous efforts have been made to identify predictive biomarkers of drug response. For example, Lindsay *et al.* provided non-linear machine learning techniques, and generated biomarkers that predict drug response [5]. David *et al.* developed a novel approach named Multivariate Organization of Combinatorial Alterations (MOCA), combining many genomic alterations into biomarkers of drug response, and found that multi-gene features have substantially higher correlation with drug response than do single-gene features [6]. It follows that methods considering the cumulative effect of many markers would make the prediction of complex phenotypes (such as drug response) more accurate [2, 7].

Based on the fact that many genes may be regarded as genomic biomarkers for drug response and one genomic biomarker may be correlated to sensitivity toward many drugs [6], a large-scale network correlating drugs and their sensitivity genes should be constructed, as it would give global clues to possible biomarker-related treatments of drug response, and network analysis would be helpful in elucidating the action of drug sensitivity.

However, it would be difficult to construct such a global drug sensitivity gene network via low-throughput biological experimental studies. One of the major concerns is that gene expression estimates, generated on different microarray platforms or even in different batches, are not always consistent, leading to irreproducible data [8]. Another factor is that publicly available data on gene expression related to drug response are relatively limited. Fortunately, these limitations could be alleviated to a great extent by the development of high-throughput experimental and bioinformatics technologies. The NCI-60 cell line panel and associated drug screens were used to pioneer the approach of linking drug sensitivity to genomic data [9]. Meanwhile, with the development of CellMiner, rapid data retrieval of genomic data along with activity reports for ∼20,000 chemical compounds across the NCI-60 was allowed [10]. Hence, we can acquire genomic data related to drug sensitivity, and build the relationship between the sensitivity genes and drug response.

In this study, we constructed a global drug-sensitivity gene network (DSGN) in which nodes represent drugs or sensitivity genes, and these are connected if the genes are related to anticancer sensitivity of the corresponding drug. We then did a series analysis of the global relationships between drugs and sensitivity genes, including the basic properties of the DSGN, shortest path analysis, Kyoto Encyclopedia of Genes and Genomes (KEGG) pathway enrichment analysis of the sensitivity genes, Anatomical Therapeutic Chemical (ATC) codes and side effects of the drugs. Through these analyses, our findings offered insight into the interplay between drugs and sensitivity genes.

## RESULTS

### Construction of the drug-sensitivity gene network (DSGN)

**Figure 1:**
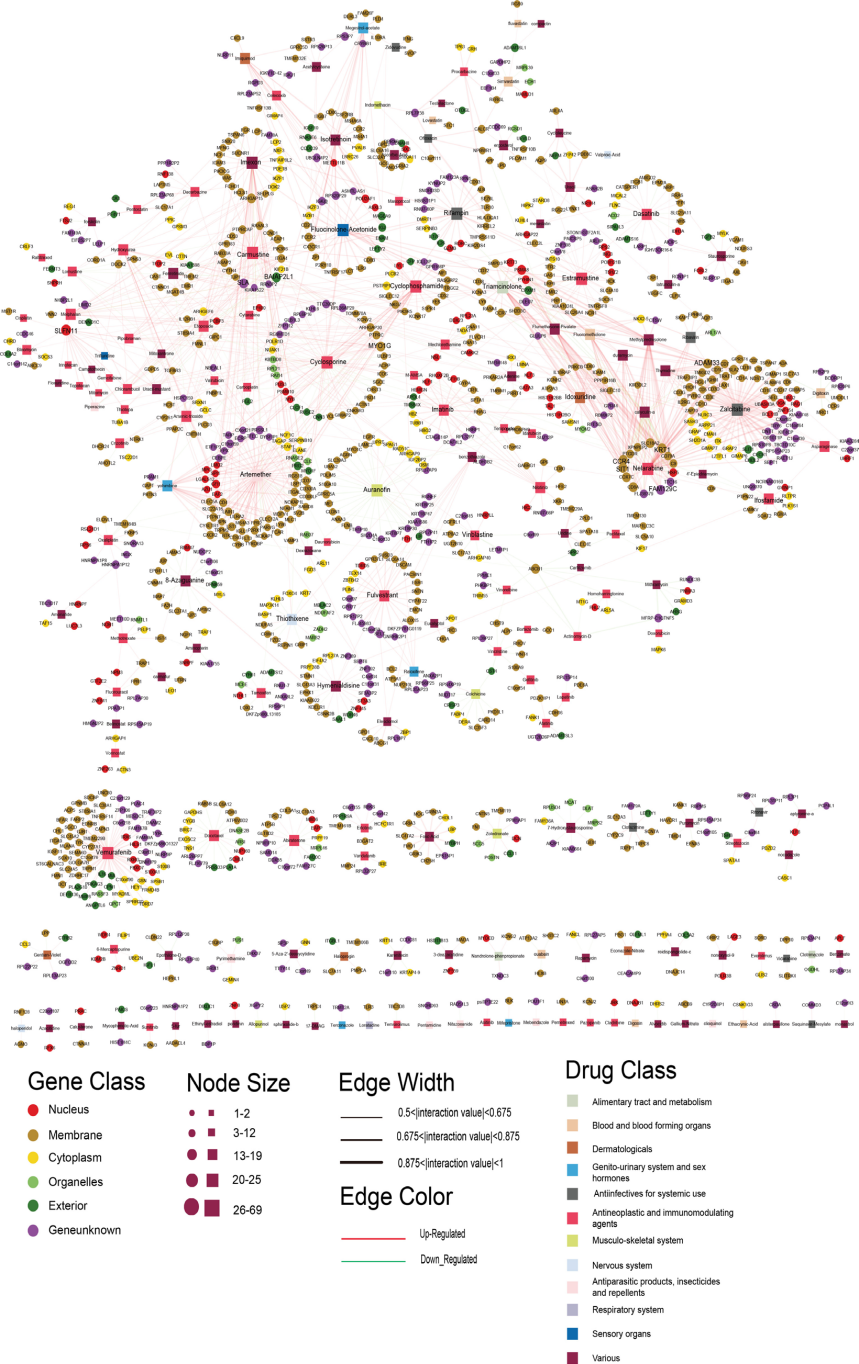
The DSGN network The rectangles and circles in the network correspond to drugs and sensitivity genes, respectively. A drug and a gene are connected by an edge if the gene is related to the anticancer sensitivity of the corresponding drug. Different colors of gene and drug nodes represent subcellular localization class of genes and ATC classification of drugs, respectively. The node size represents the degree of node.

We constructed a bipartite network consisting of two disjoint kinds of nodes. One kind of node corresponded to drugs tested in the National Cancer Institute (NCI) Developmental Therapeutics Program (DTP), and the other kind of node comprised the sensitivity genes from the profiles for the 60 cell lines of the NCI DTP drug screen [11]. A drug and a gene were connected if the gene was related to the anticancer sensitivity of the corresponding drug. To obtain the relationships between them, we used CellMiner Analysis tools (https://discover.nci.nih.gov/cellminer/) to retrieve potential associations between a drug and its sensitivity genes by calculating the Pearson correlation coefficient (PCC). Setting |PCC| ≥ 0.5 and *p*-value < 0.01, we obtained 16,694 significant drug-gene correlations, including 6477 genes and 234 drugs. Each drug was associated with up to 27.6 sensitivity genes on average. To reduce the false positive results and obtain the more significant drug-sensitivity gene relationships, we ranked the sensitivity genes for each drug according to the absolute PCC value, and retained the top 10% of sensitivity genes (see Materials and Methods). Finally, the DSGN was composed of 1646 drug-gene pairs, including 1057 genes and 181 drugs that were grouped into 12 drug classes using the ATC classification system in the DSGN (see Figure 1).

### Properties and functional mapping of the DSGN

The DSGN was composed of 1238 nodes (1057 sensitivity genes and 181 drugs) and 1646 edges (Figure 1; Supplementary Dataset 1). We examined the DSGN, and its network topological characteristics are displayed in Supplementary Figure 1. The degree distributions of the drug and gene nodes both followed power law distributions approximately with slopes of −5.45 and −6.24, respectively, and *R*^2^ = 0.27 and 0.35, respectively (Supplementary Figure 1A, 1B). Thus, the DSGN was scale-free. The degree of genes was distributed from 1 to 19. Schlafen family member 11 (*SLFN11*), with the highest degree of 19, was recently discovered as a dominant response factor of cancer cells to topoisomerase I inhibitors [12, 13]. Knockdown of SLFN11 increases chemoresistance of cancer cells to a broad range of DNA damaging agents [12, 14], and ectopic expression of SLFN11 sensitizes colon cancer cells to topoisomerase I inhibitors [15], consistent with the involvement of SLFN11 in the DNA damage response [12]. The gene with the second highest degree (12) was Src-like-adaptor (SLA), which is expressed in a variety of cell types, and it can both inhibit and activate signaling downstream of various cell surface receptors including the B cell receptor, the T cell receptor, cytokine receptors and receptor tyrosine kinases, which are important regulators of immune and cancer cell signaling [16]. SLA protein appears to be an important component in regulating signal transduction required by immune and malignant cells [16], so it was also relevant for many anticancer drugs. Meanwhile, the degree of drugs was distributed from 1 to 69, and the drug Vemurafenib, which is a B-Raf enzyme inhibitor developed for the treatment of late-stage melanoma [17], had the highest degree. Other drugs with high degrees included Zalcitabine with a degree of 68, which is a potent inhibitor of HIV replication at low concentrations, acting as a chain-terminator of viral DNA by binding to reverse transcriptase [18], and Artemether with a degree of 63, which is an antimalarial agent used to treat acute uncomplicated malaria [19].

**Figure 2:**
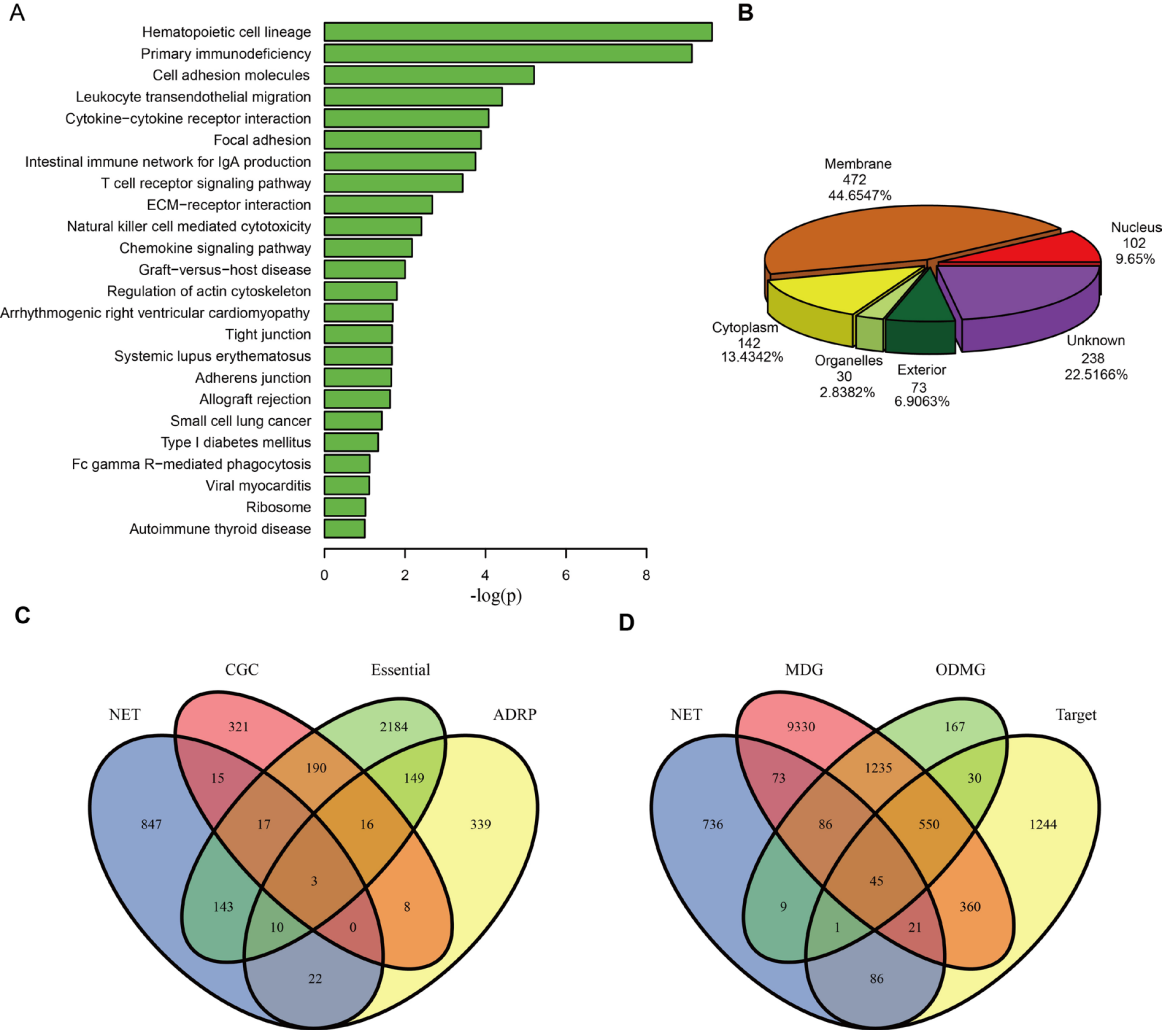
Functional annotations of the drug sensitivity genes in DSGN (**A**) KEGG pathways enriched by sensitivity genes. (**B**) Pie chart of 1057 sensitivity genes categorized into six groups according to subcellular localization: membrane, cytoplasm, organelles, nucleus, exterior and genes that did not belong to any group above (unknown). (**C**) The Venn diagram of overlaps among the 1057 sensitivity genes (NET), 571 Cancer Gene Census genes (CGC), 2712 essential genes (Essential) and 547 adverse drug reaction-associated proteins (ADRP). (**D**) The Venn diagram of overlaps among the 1057 sensitivity genes (NET), 11,700 Mendelian disease genes (MDG), 2123 orphan disease-causing mutant genes (ODMG) and 2354 drug target genes (Target).

To investigate the biological functions of drug sensitivity genes in the DSGN, we employed functional analysis of the corresponding genes. We implemented KEGG pathway enrichment by using DAVID [20], and found 24 pathways were significantly enriched (*p*-value < 0.01) (Table 1, Figure 2A). The hematopoietic cell lineage (hsa04640) pathway was the most significantly enriched pathway, and it is significantly associated with pediatric acute lymphoblastic leukemia [21]. The primary immunodeficiency (hsa05340) pathway was also a significantly enriched pathway, which might be associated with acute myeloid leukemia (AML) development [22].

**Table 1:** Significantly enriched pathways

	Term	*P* Value	FDR
1	hsa04640:Hematopoietic cell lineage	2.37E-10	2.86E-07
2	hsa05340:Primary immunodeficiency	7.48E-10	9.03E-07
3	hsa04514:Cell adhesion molecules (CAMs)	6.22E-06	0.007512
4	hsa04670:Leukocyte transendothelial migration	3.85E-05	0.046522
5	hsa04060:Cytokine-cytokine receptor interaction	8.34E-05	0.100689
6	hsa04510:Focal adhesion	1.28E-04	0.154968
7	hsa04672:Intestinal immune network for IgA production	1.78E-04	0.214605
8	hsa04660:T cell receptor signaling pathway	3.70E-04	0.445435
9	hsa04512:ECM-receptor interaction	0.002096	2.501833
10	hsa04650:Natural killer cell mediated cytotoxicity	0.003898	4.606564
11	hsa04062:Chemokine signaling pathway	0.00668	7.773949
12	hsa05332:Graft-versus-host disease	0.009976	11.40227
13	hsa04810:Regulation of actin cytoskeleton	0.015899	17.59498
14	hsa05412:Arrhythmogenic right ventricular cardiomyopathy (ARVC)	0.020163	21.80382
15	hsa04530:Tight junction	0.02086	22.47301
16	hsa05322:Systemic lupus erythematosus	0.021074	22.67742
17	hsa04520:Adherens junction	0.021923	23.48353
18	hsa05330:Allograft rejection	0.023549	25.00512
19	hsa05222:Small cell lung cancer	0.037487	36.95679
20	hsa04940:Type I diabetes mellitus	0.046316	43.59542
21	hsa04666:Fc gamma R-mediated phagocytosis	0.075082	61.03248
22	hsa05416:Viral myocarditis	0.076974	61.98434
23	hsa03010:Ribosome	0.096177	70.50742
24	hsa05320:Autoimmune thyroid disease	0.099817	71.91028

We then characterized the sensitivity genes in the DSGN by categorizing them into six groups according to subcellular localization: membrane, cytoplasm, organelles, nucleus, exterior and genes that did not belong to any group above (unknown). Figure 2B shows the distribution of these sensitivity genes among the six groups. We found that up to 472 (44.65%) genes belonged to the membrane group, which indicates that these genes are likely to participate in the processes of cell membrane function, such as the transport process and membrane receptor recognition process.

We also compared the 1057 sensitivity genes with each of the following gene sets: 571 Cancer Gene Census genes (CGC), 2712 essential genes (Essential), 547 adverse drug reaction-associated proteins (ADRPs), 11,700 Mendelian disease genes (MDGs), 2123 orphan disease-causing mutant genes (ODMGs) and 2354 drug target genes (Target). Figure 2C and 2D show the overlaps between the sensitivity genes and the other six gene sets. Although the number of overlaps is small, we still found that sensitivity genes significantly overlapped with CGC (*p* = 0.00546), Essential (*p* = 7.9e-10), ADRPs (*p* = 0.002763) and ODMGs (*p* = 9.792813e-10) (hyper geometric distribution test), indicating that many sensitivity genes are possibly cancer genes, essential genes, adverse drug reaction-associated proteins and orphan disease-causing mutant genes.

To further investigate the drugs and sensitivity genes in the DSGN, we generated two biologically relevant network projections, a "drugs–drug network" (DDN) and a "sensitivity gene network" (SGN) from the DSGN bipartite network.

### Functional characteristics of the DDN

In the DDN (composed of 125 nodes and 564 edges), the nodes represented the drugs, and two drugs were connected to each other if they were connected to at least one common gene in the DSGN (Supplementary Figure 2). Some researchers have indicated that drugs that bind to similar proteins tend to have similar effects and pharmacological properties [23–25]. Thus arose the question whether, if two drugs share the same sensitivity genes, they tend to cause the same therapeutic or side effects.

**Figure 3:**
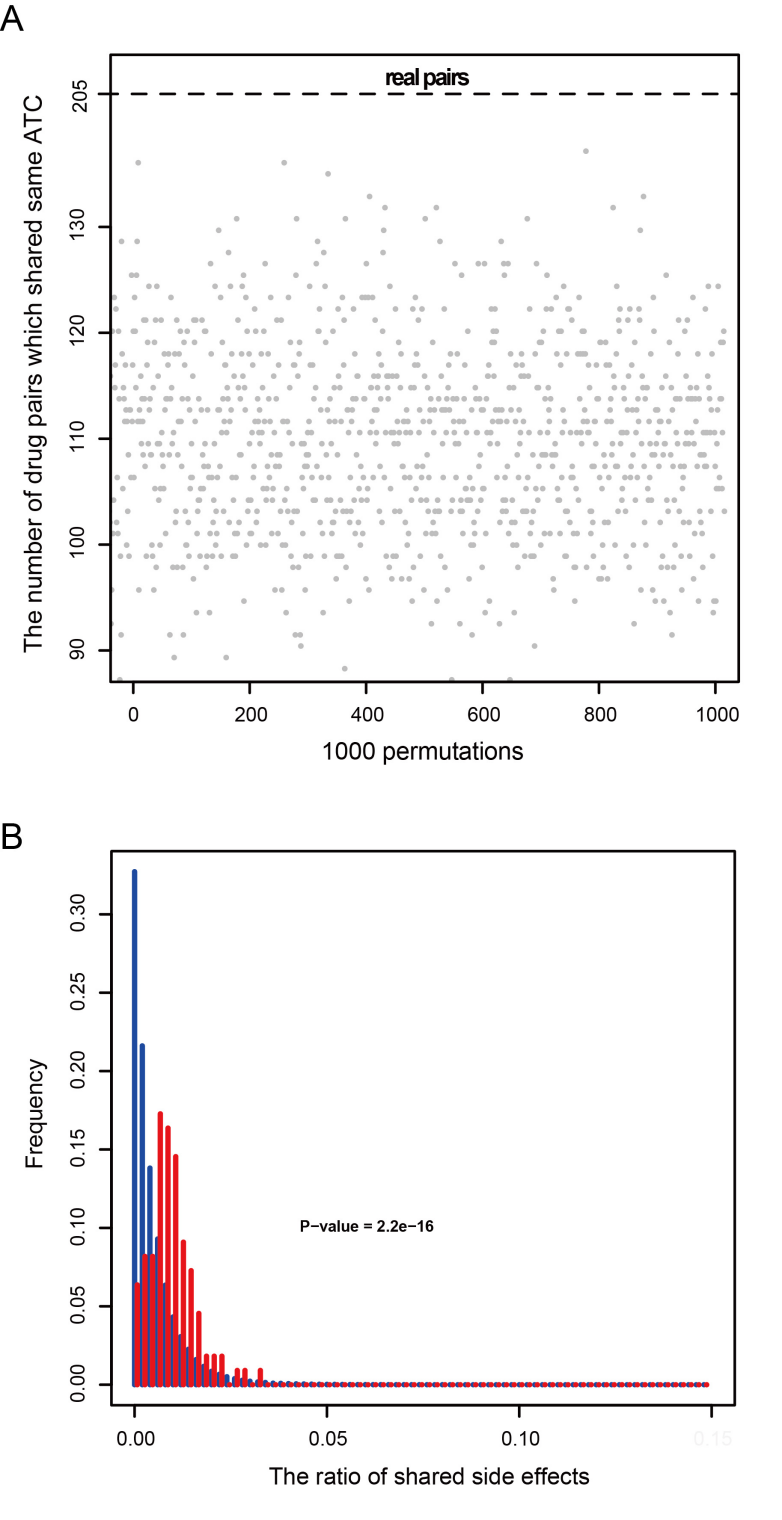
Characteristics of drug pairs that have the same sensitivity genes (**A**) 205 drug pairs that have the same sensitivity genes share the same ATC classification, compared to 1000 times permutations. (**B**) The proportion of shared side effects by drug pairs that have the same sensitivity genes (red), compared to the proportion of shared side effects among the total drug pairs in the SIDER database (blue).

To investigate this, we obtained the ATC classifications of 125 drugs and found that 205 drug-drug pairs out of 564 edges in the DDN belonged to the same ATC classification. We then generated 564 randomized drug pairs 1000 times. We found that there were no times when the number of randomized drug pairs that shared the same ATC classification was more than 205, suggesting that connected pairs tend to share the same ATC classification (*p*-value < 0.001, Figure 3A). Taking Chlorambucil and Carmustine for example, Chlorambucil is a nitrogen mustard alkylating agent used as an antineoplastic agent for the treatment of various malignant and nonmalignant diseases, while Carmustine is a cell-cycle phase nonspecific alkylating antineoplastic agent used for the treatment of brain tumors, multiple myeloma, Hodgkin’s disease and non-Hodgkin’s lymphomas. The two drugs were connected by some sensitivity genes, and they belong to the same ATC code L (antineoplastic and immunomodulating agents).

Then we downloaded the public and accurate side effect records from the SIDER database that includes 997 drugs corresponding to 4492 side effects. In the DDN, there were 71 drugs that were also recorded in the SIDER database and these drugs formed 110 unique connected drug pairs that share the same side effect (Figure 3B). In the SIDER database, some side effects, such as dizziness and nausea, were caused by most drugs. To improve the specificity of the similarity of drug pairs, we calculated the number of side effects shared by drug pairs rather than the number of drug pairs that shared the same side effects. We found that the number of side effects shared by connected drug pairs of the DDN was significantly higher than the number of side effects shared by total drug pairs in the SIDER database (*p*-value = 2.2^*^e-16, Wilcoxon rank sum test). These results suggested that two drugs sharing the same sensitivity genes in the DDN tend to cause the same side effects.

### Functional characteristics of the SGN

We also constructed the (SGN), in which two genes are related by anticancer sensitivity toward the same drug in the DSGN. SGN was composed of 1033 nodes and 15,224 edges (Supplementary Figure 3).

**Figure 4:**
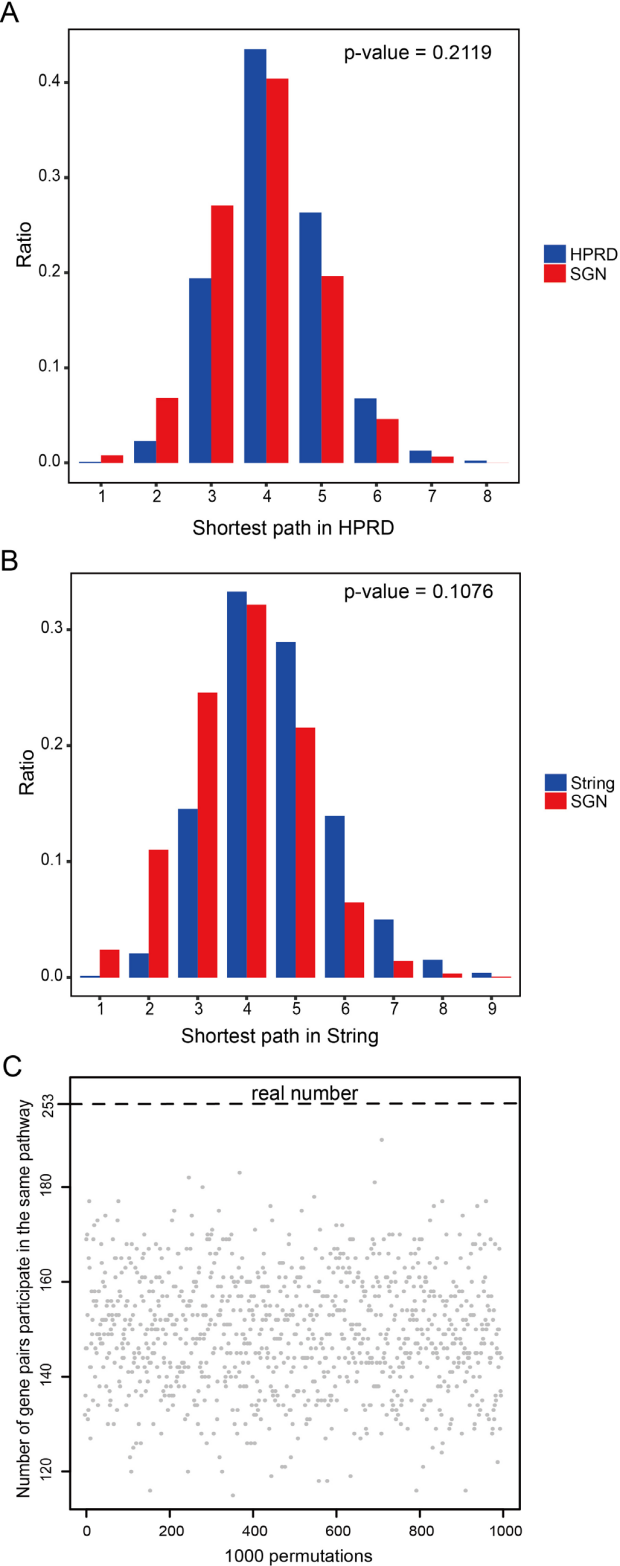
Functional characterizations of sensitivity genes belonging to the same drug (**A**) Distribution of the shortest paths of sensitivity genes belonging to the same drug in the HPRD network (red), compared to the shortest paths among all nodes in the HPRD (blue). (**B**) Distribution of the shortest paths of sensitivity genes belonging to the same drug in the STRING network (red), compared to the shortest paths among all nodes in the STRING (blue). (**C**) 253 gene pairs participated in the same pathway, compared to 1000 permutations.

Yildirim *et al.* indicated that targets of a single drug tend to be in the same module of a network of physical protein-protein interactions (PPIs) and participate in the same molecular complex or cellular pathway [26]. Thus arose the problem whether the sensitivity genes of the same drug also display this feature. First, we investigated the distribution of sensitivity genes belonging to the same drug by calculating the shortest path between sensitivity genes in the Human Protein Reference Database (HPRD) [27] and STRING [28] network using the classical Dijkstra algorithm. We observed a strong enrichment in the regions of first, second and third neighbors compared with the distribution of the shortest path between all nodes in the HPRD and STRING PPI network (Figure 4A, 4B), showing a bias toward clustering of the sensitivity genes of the same drug in the SGN network.

We then investigated whether the sensitivity gene pairs of the same drug tend to participate in the same pathway. We found that there were 253 connected gene pairs in the SGN engaging in 39 pathways. We then randomized the gene pairs 1000 times, and we found that there were no times when the number of randomized gene pairs that engaged in the same pathway was more than 253, suggesting that connected pairs tend to participate in the same pathway (*p*-value < 0.001, Figure 4C).

### Correlation between a sensitivity gene and drug target gene in the context of the cellular network

**Figure 5:**
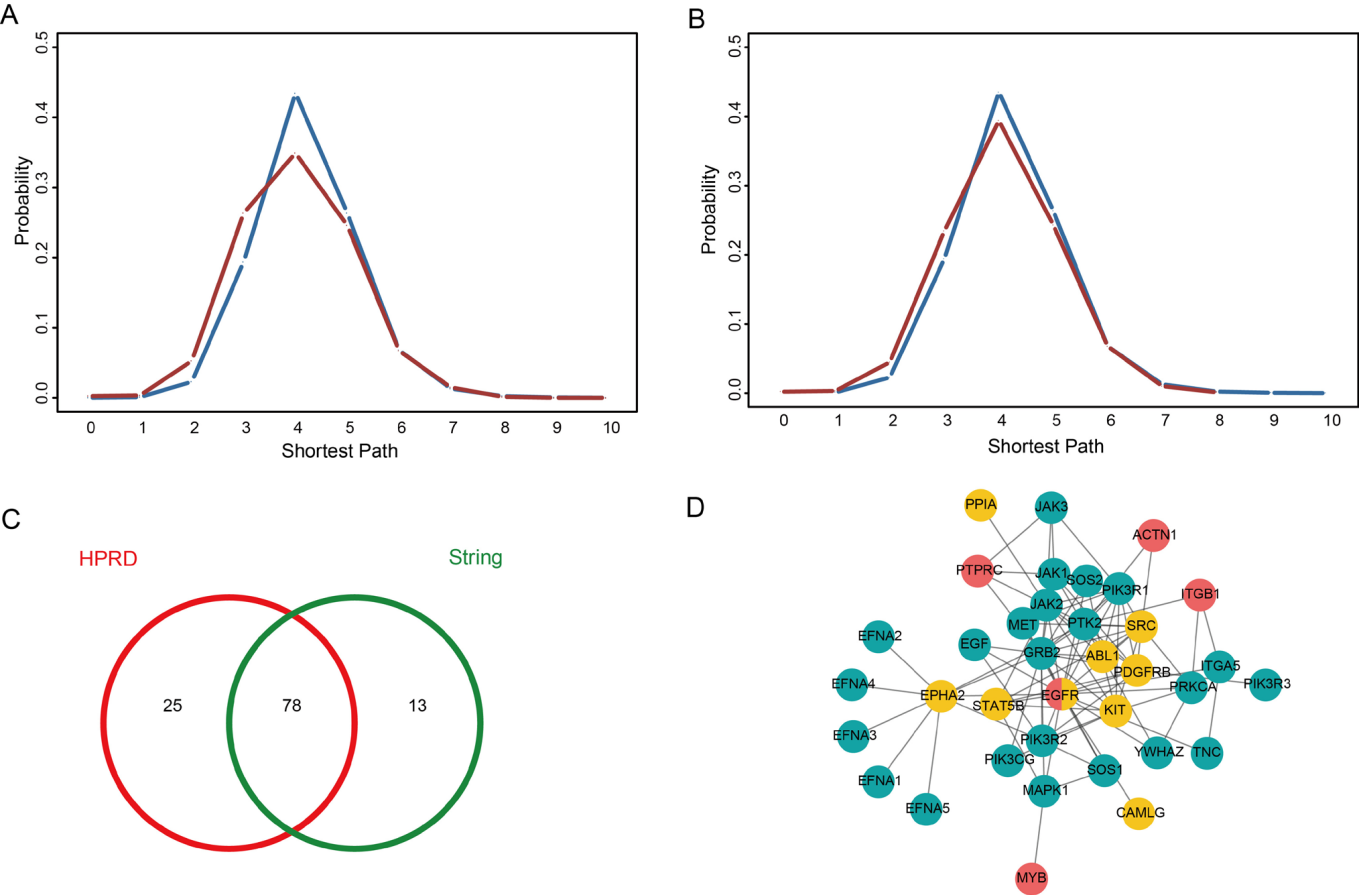
The relationships between a sensitivity gene and a target gene for the same drug (**A**) Distribution of the shortest paths between a sensitivity gene and a target gene for the same drug in the HPRD network (red), compared to the shortest path distribution for all nodes in the HPRD (blue). (**B**) Distribution of the shortest paths between a sensitivity gene and a target gene for the same drug in the STRING network (red), compared to the shortest path distribution for all nodes in the STRING network (blue). (**C**) Among the shortest paths between sensitivity genes and target genes that are less than 4, 103 drugs are in the HPRD network and 91 drugs are in the STRING network, of which 78 drugs overlap between the two PPI networks. (**D**) Subnetwork of epidermal growth factor receptor (EGFR) and its neighbor nodes from the HPRD network; red corresponds to sensitivity genes, yellow corresponds to target genes, and green corresponds to enzymes in the PI3K/Akt signaling pathway.

Tumor-targeted delivery of compounds to the site of malignancy allows for enhanced cellular uptake and increased therapeutic effects, so the development of targeted delivery greatly improved anticancer therapeutic research [29]. Meanwhile, molecular biomarkers have been widely used to predict anticancer drug response. Thus arose the question whether the sensitivity genes and drug target genes are functionally related, and we explored the mechanism of action of sensitivity genes by investigating the relationships between sensitivity genes and target genes. To further investigate the functions of sensitivity genes, we also analyzed the relationships between the sensitivity genes and the target genes of the same drug based on the PPI network. We obtained the drug targets from the DrugBank database (http://www.drugbank.ca), which is a richly annotated database of drug and drug target information [30]. All of the sensitivity genes and target genes were mapped into the PPI network (HPRD and STRING). In the PPI network, we calculated the shortest path between a sensitivity gene and target gene for each drug, and we found that the shortest paths followed a normal distribution, the shortest paths being between 0 and 10 (0 represented the sensitivity gene and the target gene being the same) and the average of the shortest paths being 4.04 for HPRD (4.07 for STRING). Then we compared the distribution of the above shortest paths with that of all nodes in the HRPD (or STRING). We found that the distribution of the shortest paths between all nodes in the PPI network was significantly larger than the distribution of the shortest paths between sensitivity genes and target genes for the same drug (Figure 5A, 5B). Thus we concluded that the sensitivity gene and the target gene of the same drug are densely connected in the PPI network, which indicates that they are functionally related. Besides, we found 103 drugs for which the shortest path between the sensitivity gene and the target gene was less than 4 in the HPRD network and 91 drugs in the STRING network, of which 78 drugs overlapped between the two PPI networks (Figure 5C), which further indicated the robustness of our results.

To further demonstrate the mechanism underlying the interaction between the sensitivity gene and the target gene, we selected epidermal growth factor receptor (EGFR) as an example. ErbB2/EGFR inhibitor is an important type of anticancer drug (e.g. Lapatinib and Varlitinib) and its gene is also a sensitivity gene in our DSGN. Tetsu *et al.* reported that inhibiting EGFR could evoke innate drug resistance in lung cancer cells by preventing Akt activity and thus inactivating Ets-1 function [31]. The expression of EPH receptor A2 (EPHA2) is activated by EGFR and EGFRvIII in the human cancer cell lines [32]. EPHA2 is also the target of Dasatinib. Interestingly, the combination of Dasatinib and Gefitinib (an EGFR inhibitor) presents anti-tumor properties that are superior to those of platinum-based combinations, indicating that this combination may be a promising new treatment modality to be tested in the clinic [33]. We extracted a subnetwork of epidermal growth factor receptor (EGFR) gene and its neighbor nodes from the HPRD network (Figure 5D). Then we found that some genes in this subnetwork (for example, PIK3R1, PIK3R3 and MET) are involved in the PI3K/Akt signaling pathway and some genes (JAK1, JAK2 and STAT5B) are involved in the JAK/STAT signaling pathway. PI3K/Akt was overexpressed and activated in cancer cells and was found to induce chemoresistance in various cancers [34–38]. For example, the PI3K/Akt pathway was found to be related to multidrug resistance in gastric cancer cells [39]. In addition, O’Gorman *et al.* found PI3-kinase inhibition significantly increased sensitivity in HL60 human leukemia cells [40]. Meanwhile, the increased JAK/STAT signaling and enhanced interference with aerobic glycolysis and autophagy are associated with resistance to Afatinib [41]. Also, the crosstalk involving PI3K/Akt and JAK/STAT pathways was related to resistance to Sorafenib, an oral multikinase inhibitor [42].

## DISCUSSION AND CONCLUSIONS

One of the biggest challenges associated with cancer chemotherapy resistance is discovering the unknown underlying mechanisms of drug sensitivity. Insight into the mechanisms of drug sensitivity is critical for effective treatment strategies in drug development. With the development of high-throughput technology, some high-quality data made it possible to reveal the potential mechanism underlying drug sensitivity. NCI-60 data assessed gene expression profiles in 60 human cancer cell lines and characterized drug activities by treatment with more than 70,000 different compounds. CellMiner provided a rapid data retrieval of genomic data along with activity reports for ∼20,000 chemical compounds across the NCI-60. They pioneered the approach of linking drug sensitivity to genomic data by the development of high-throughput experimental and bioinformatics technologies.

We constructed a DSGN using CellMiner Analysis tools to retrieve potential associations between a drug and its sensitivity genes in the NCI-60 cell line panel.

Network analysis of the DSGN offered insight into the interplay between drugs and sensitivity genes. We noticed that two drugs sharing the same sensitivity genes tended to share the same ATC classification and tended to cause the same side effects. Interestingly, we investigated the biological functions of the drug sensitivity genes, and found significantly enriched pathways were associated with cancer occurrence and development. Furthermore, compared to other functional gene sets, the sensitivity genes tended to be cancer genes, essential genes, adverse drug reaction-associated protein genes and orphan disease-causing mutant genes.

We also investigated the different sensitivity genes of the same drug. We found the sensitivity genes tended to cluster together in the PPI network when we calculated the shortest path. Besides, by implementing pathway enrichment analysis, we found sensitivity gene pairs that belonged to the same drug tended to participate in the same pathway. That indicated that sensitivity genes of the same drug tended to engage in the same module of the PPI network and participate in the same biological functions. In view of the fact that anticancer therapeutic research is significantly associated with targeted delivery and molecular biomarkers, we investigated the relationships between the sensitivity genes and target genes based on the PPI network by calculating the shortest path between them, and we found that the sensitivity gene and target gene of the same drug were densely connected, indicating that they are functionally related. Interestingly, we found that the drug combination Dasatinib and Gefitinib could be inferred from the interaction of their targets, *EPHA2* and *EGFR*, suggesting that targeting sensitivity genes/proteins may be a potential strategy against drug resistance.

We also noticed that there were some limitations of our current study. Firstly, compared to tens of thousands of drugs, DSGN only include 181 FDA-approved anticancer small molecular drugs. It would be improved by the development of high-throughput experiments and pharmacogenomics. Another limitation of our study is the data source of DSGN was relatively simple. Besides NCI-60 dataset, integrating more drug-affected gene expression profiles of other resources and literatures will alleviate this limitation and complement DSGN. Although these data sets and the methodology are far from complete, our network analyses still provide statistically significant characteristics of the relationships between drugs and sensitivity genes.

In conclusion, we constructed a drug-sensitivity gene network (DSGN) based on the potential relationships between drugs and their sensitivity genes in the NCI-60 cell line panel, which contained 181 drugs, 1057 sensitivity genes and 1646 associations. Then we did a series of analysis to look for insights into the global relationships between drugs and their sensitivity genes, such as network analysis of DSGN, shortest path analysis between sensitivity gene and target gene, KEGG pathway enrichment analysis of sensitivity genes, ATC and side effects analysis of drugs, and so on. At last, our analyses provide statically significant characteristics of the relationships between drugs and sensitivity genes and help to gain insight into the mechanism of drug sensitivity.

## MATERIALS AND METHODS

### Generating the DSGN

We collected drugs and sensitivity genes from the NCI-60 cancer cell line database. The NCI-60 cancer cell line database is a large-scale information set with multiple genomic and drug response platforms. We retrieved potential associations between the drug activity and the expression levels of mRNAs by CellMiner, which is a powerful platform that allows rapid data retrieval of transcripts for genes along with activity reports for chemical compounds. The CellMiner provides ‘NCI-60 Analysis Tools’ to study the relationships between the mRNA expression and the 50% growth inhibitory concentration (GI50) values of drugs by calculating the Pearson correlation coefficient (PCC) between them. Firstly, we selected U.S. food and drug administration (FDA)-approved drugs and clinical trials drugs, and filtered out drugs not in the DrugBank database. Then we followed the steps to retrieve the correlations: (i) in the ‘NCI-60 Analysis Tools’ page, click ‘Pattern comparison’ and ‘Drug NSC#’ option in Step 1 section; (ii) input the drug NSC ID in Step 2 section; (iii) enter e-mail address and CellMiner would send the result documents of Pearson correlations between all genes and each input drug; (iv) integrated all drug files together. Assigning 0.5 as the PCC threshold, we obtained 16,694 correlations of drug-gene pairs, encompassing 6477 genes and 234 drugs, each drug was associated with up to 27.6 sensitivity genes on average. Then we did some dealing steps to reduce the false positive results and obtain the more significant drug-sensitivity gene relationships. For each drug: (i) we ranked the drug-gene pairs according to the absolute PCC value in descending order, and counted the number of sensitivity genes, and rounded this number up to 10-fold value rd (e.g. if the drug was related with 32 sensitivity genes, then rd = 40); (ii) we retained the top 10%*rd of the ranked drug-gene pairs (e.g. if rd = 40, then we retained top 4 drug-sensitivity gene pairs); (iii) we mapped NSC ID to the drug names. Finally, we obtained 1646 drug-gene pairs, encompassing 1057 genes and 181 drugs.

### SIDER database

We obtained drug side effects data from a public computer-readable side effect resource, the side effect resource (SIDER) [43], which is freely available for academic research on the website http://sideeffects.embl.de. We collected 997 drugs corresponding to 4492 side effect terms. In the DSGN, 71 drugs were recorded in the SIDER database.

### Different gene sets

#### CGC genes

The cancer genes are genes for which mutations have been causally implicated in cancer. We downloaded 571 cancer genes from the CGC (http://cancer.sanger.ac.uk/cancergenome/projects/census/), which is an ongoing effort to catalogue these cancer genes.

#### Essential genes

Essential genes are crucial in the study of the robustness of biological systems and effective drug target identification, while knockouts of them would result in a lack of cell viability or embryonic lethality. We collected 2721 essential genes from the Online GEne Essentiality (OGEE) database [44].

#### ADRPs

ADRPs are proteins that mediate adverse drug reactions or toxicity by binding to drugs or their reactive metabolites. We collected 547 ADRPs from Chen’s previously published work [45].

#### MDGs

We collected 11,700 MDGs from the Online Mendelian Inheritance in Man database [46], which is a comprehensive, authoritative and timely knowledge base of human genes and genetic disorders compiled to support human genetics research and education and the practice of clinical genetics.

#### ODMGs

An orphan disease is a disease that affects fewer than 200,000 inhabitants, which is equivalent to approximately 6.5 patients per 10,000 inhabitants, and ODMGs are orphan disease-causing mutant genes. We collected 2123 ODMGs from Zhang’s previously published work [47].

#### Drug target data source

The drug-target associations were downloaded from the DrugBank database [48]. We obtained target genes of 6905 drugs. Duplicated target genes without Entrez ID were excluded, and we collected 2354 drug target genes.

### Extraction of subnetwork related to sensitivity gene and drug target gene

We selected epidermal growth factor receptor (EGFR) as example to study the mechanism underlying the interaction between the sensitivity gene and the target gene. EGFR gene and its first neighbor nodes were extracted from the HPRD to construct a subnetwork.

## SUPPLEMENTARY MATERIALS FIGURES




