# Supplementary material for: Global view of a drug-sensitivity gene network
Source: Oncotarget. 2017 Dec 14;9(3):3254–66. doi: 10.18632/oncotarget.23229 (PMC5790461; doi:10.18632/oncotarget.23229)
Supplement: Supplementary file 1 [file oncotarget-09-3254-s001.pdf]

Global view of a drug-sensitivity gene network

SUPPLEMENTARY MATERIALS

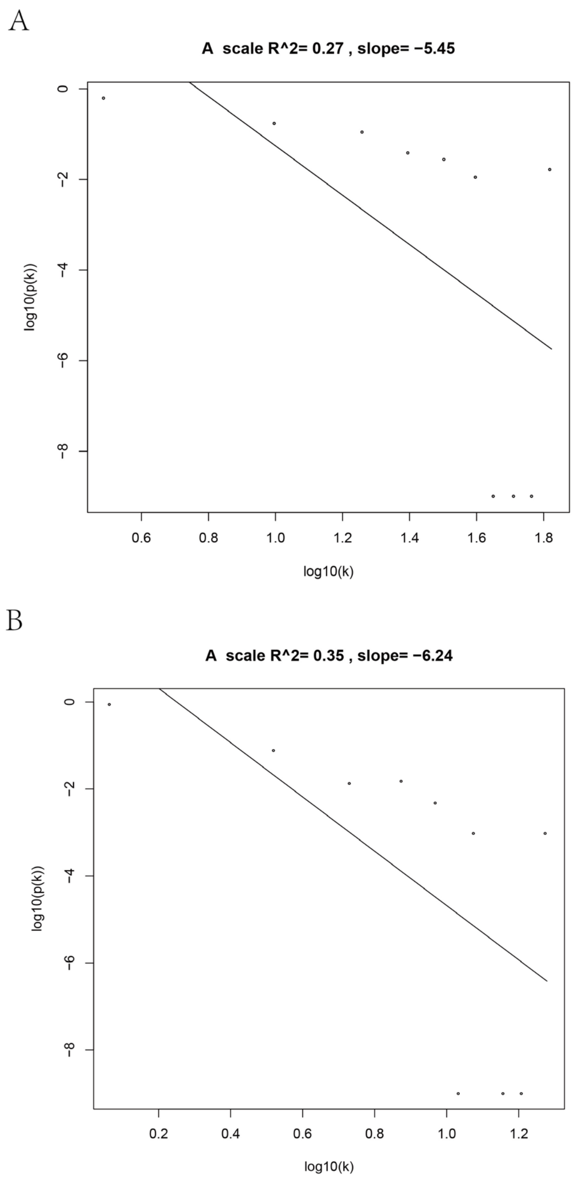

Supplementary Figure 1: The degree distribution of the drug and gene nodes in DSGN. (**A**) The degree distribution of the drugs. (**B**) The degree distribution of the sensitivity genes.

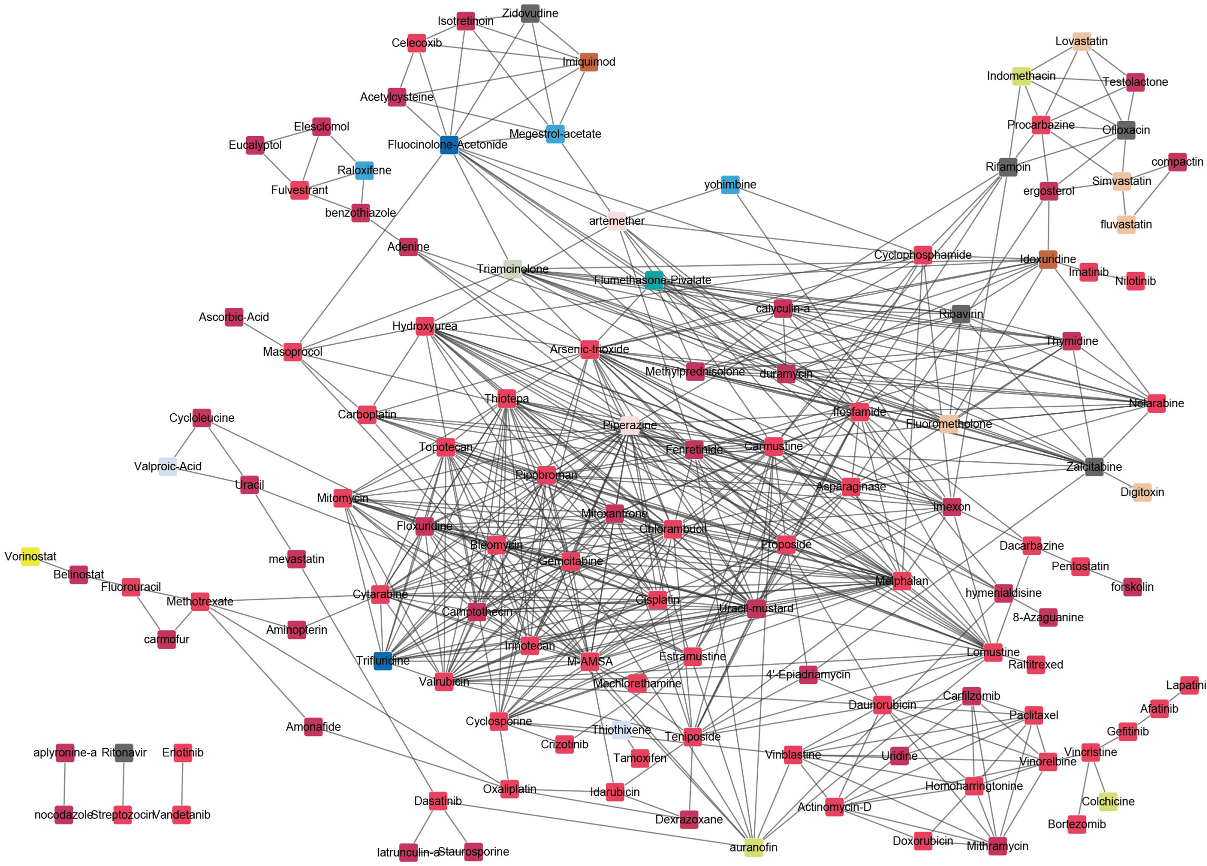

Supplementary Figure 2: The "drug-drug network" (DDN). Nodes correspond to drugs, and the color corresponds to the ATC classification to which the drug belongs.

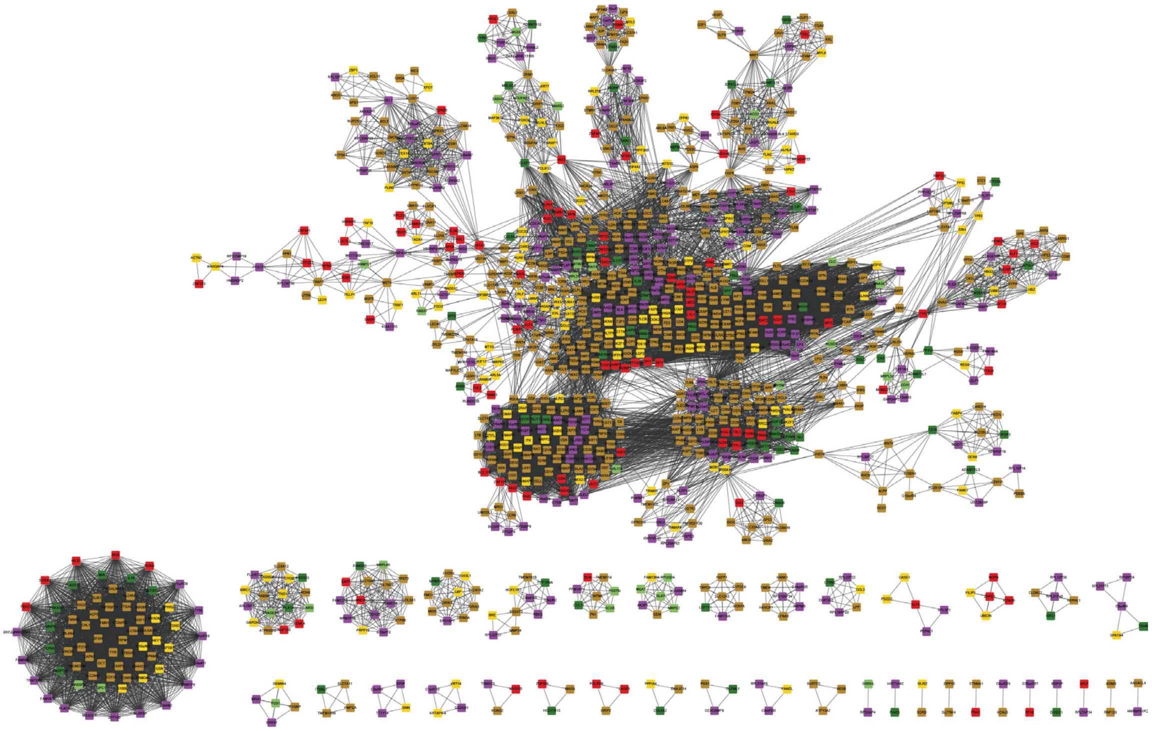

Supplementary Figure 3: The "sensitivity gene network" (SGN). Nodes correspond to sensitivity genes, and the color corresponds to the subcellular localization to which the gene belongs.

Supplementary Dataset 1: Relationships of drugs and sensitivity genes in DSGN. See Supplementary_Dataset_1
